# Targeting PI3Kγ in cancer

**DOI:** 10.1016/j.trecan.2025.01.008

**Published:** 2025-02-12

**Authors:** Giuliana P. Mognol, Anghesom Ghebremedhin, Judith A. Varner

**Affiliations:** 1 Moores Cancer Center, University of California, San Diego, La Jolla, CA 92093-0819, USA; 2 Department of Pathology, University of California, San Diego, La Jolla, CA 92093-0819, USA

## Abstract

The phosphoinositide 3-kinases (PI3Ks) have been the focus of a significant body of cancer research since their discovery nearly 40 years ago. These lipid kinases are now known to play central roles in cancer cell proliferation, survival, migration, metabolism, and immunity and serve as the target of numerous investigational and approved therapeutics. One of these kinases, the unique class IB PI3Kγ, which is highly expressed in myeloid lineage cells and myeloid leukemias, plays prominent roles in tumor immune suppression. Inhibition of this kinase has promoted improved antitumor immune responses in recent solid tumor preclinical studies and clinical trials. New studies also identify this kinase as a driver of acute myeloid leukemia self-renewal and as a new target for the treatment of aggressive leukemias.

## PI3Kγ: a new target for solid and liquid tumor therapy

**Figure 1.**
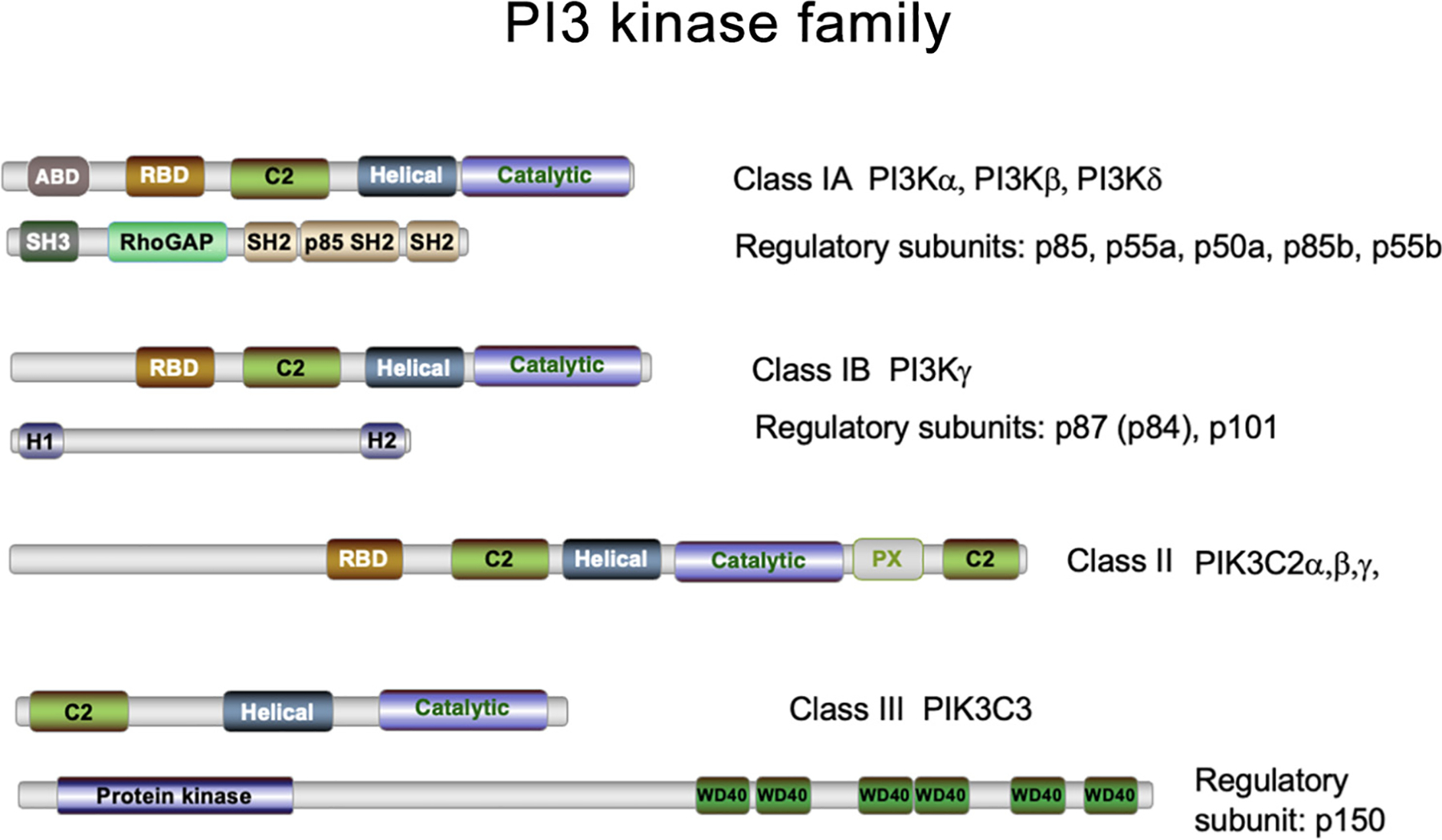
Structure of the phosphoinositide 3-kinase (PI3K) family. The PI3K family of inositol lipid kinases is composed of three structurally and functionally related subfamilies: class I, class II, and class III. Each PI3K enzyme contains a structurally related catalytic domain, an alpha-helical domain, and a calcium-dependent phospholipid binding motif (C2). Classes I and II PI3Ks also contain a Ras-binding domain (RBD). Some catalytic subunits contain an accessory binding domain (ABD) as well. Regulatory subunits localize PI3Ks to subcellular compartments and modulate catalytic activity. Composed of two subclasses, class IA and class IB, the class I family phosphorylates phosphatidylinositol-4,5-bisphosphate at the 3′ position to create phosphatidylinositol-3,4,5-phosphate [PtdIns(3,4,5)P3] at the plasma membrane, which nucleates numerous signaling pathways associated with cell adhesion, migration, nutrient sensing, proliferation, and survival. The class II family of PI3Ks phosphorylates two phosphoinositides, PI(3)P and PI(3,4)P2, and plays roles in endocytosis and mitosis. The class III PI3K regulates intracellular trafficking during autophagy, endocytosis, and phagocytosis.

First described in 1985 as a family of lipid kinases and in 1988 as a family of phosphoinositide 3-kinases (**PI3Ks; see** Glossary), the PI3K family of lipid kinases phosphorylate phosphatidylinositol and are now known to control metabolism, cell proliferation, migration, oncogenesis, vascular permeability, and immunity [1–5]. The initial discoveries of these kinases led to the identification of three classes of PI3Ks, I, II, and III, each with distinct subcellular localizations, functions, targets, and cell type expression (Figure 1). The four **class I PI3Ks**, the most well-known of this family of lipid kinases, are heterodimers composed of one structurally related catalytic subunit (p110α, p110β, p110γ, or p110δ) and one regulatory subunit. Class I PI3Ks are divided into two subfamilies on the basis of structural differences in catalytic domains and the regulatory subunits associated with them. Class IA consists of the catalytic subunits p110α, p110β, and p110δ and the regulatory subunits p85α, p55α, p50α, p85β, and p55γ, and class IB consists of one structurally distinct catalytic subunit (p110γ) and two unique regulatory subunits (p101 and p84/87) [5]. Class I regulatory subunits play essential roles in controlling PI3K protein subcellular localization and in maintaining catalytic subunits in a stable, inactive conformation. As a group, class I PI3Ks can be activated by cell surface tyrosine kinase receptors, G protein–coupled receptors (GPCRs), and Ras family proteins. These kinases relay signals from the cell surface to intracellular organelles and the nucleus to impact cell proliferation, survival, gene expression, and/or metabolism. Of the class I PI3Ks, the class IA PI3Kα is most well-known as a frequently mutated oncogene and a key regulator of insulin signaling, whereas two class I PI3Ks, δ and γ, are critically important in the immune system. The enzymatic activities of these kinases are opposed by the tumor suppressor phosphatase and tensin homolog (PTEN), which dephosphorylates phosphatidylinositol-3,4,5-trisphosphate (PIP_3_); a balance of these two proteins modulates cell behaviors such as cell migration and survival [6].

Of the four class I PI3Ks, the class 1B **PI3Kγ** isoform plays unique roles in cancer immunology. PI3Kγ kinase is primarily expressed in myeloid lineage cells, including monocytes, neutrophils, macrophages, dendritic cells, and mast cells; it is also highly expressed in endothelial cells and occasionally in tumor cells [7–9]. PI3Kγ plays key roles in the **trafficking** and movement of myeloid cells into infected, injured, and oncogenic tissues and in the control of immune-suppressive, wound-healing–associated gene expression in dendritic cells, granulocytes, macrophages, lipid-associated macrophages, and myeloid-derived suppressor cells (MDSCs) [7,10]. Loss of PI3Kγ expression or function strongly reduces tumor macrophage content and blocks tumor **immune suppression** [7,10]. PI3Kγ also regulates neutrophil extracellular trap formation (**NETosis**), oxidative bursts, and phagocytosis in myeloid cells [11–15]. It negatively regulates endothelial cell–cell junctions, promoting vascular leak while simultaneously promoting myeloid cell entry into tissues [16].

**Figure 2.**
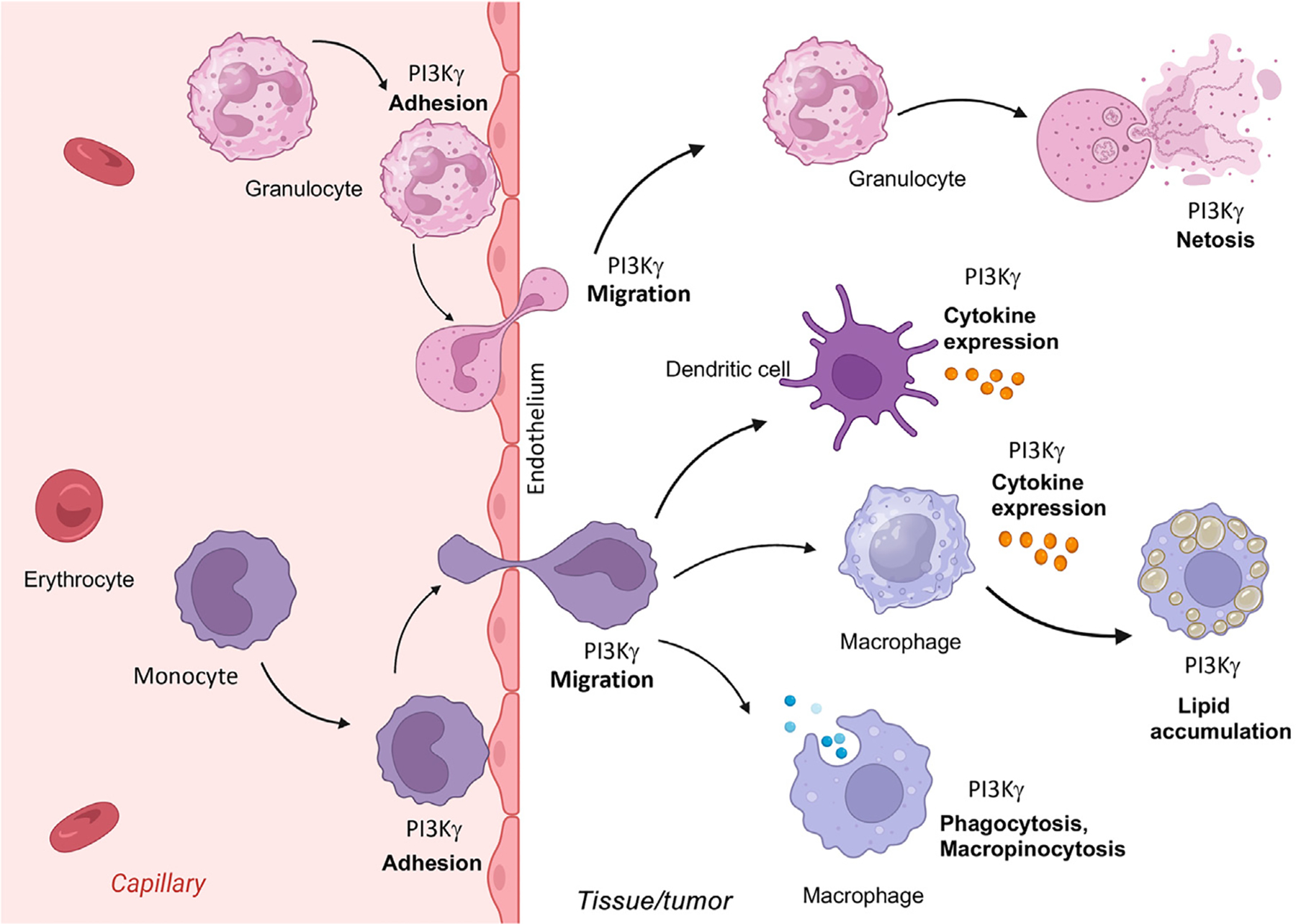
Key figure. Phosphoinositide 3-kinase-γ (PI3Kγ) roles in innate immunity The class IB PI3Kγ isoform controls several key properties of myeloid cells during inflammation. This kinase controls the trafficking and movement of myeloid cells into infected, injured, and oncogenic tissues by facilitating rapid adhesion of myeloid cells, including monocytes (purple) and granulocytes (pink), to endothelium in capillaries in diseased tissues. PI3Kγ then regulates myeloid cell migration in tissues, dendritic cell and macrophage cytokine expression, and macrophage-mediated macropinocytosis and phagocytosis as well as macrophage lipid accumulation. PI3Kγ also regulates neutrophil extracellular trap formation (NETosis).

PI3Kγ is activated by GPCRs, such as CCR2 and CXCR4 [15]. In addition, some studies have found that PI3Kγ can be activated by receptor tyrosine kinases (RTKs), such as colony-stimulating factor 1 receptor (CSF1R) and VEGFR1/2 (vascular endothelial growth factor receptor 1/2), in a Ras-dependent manner [7]. PI3Kγ can also be directly activated by Ras proteins [7]. Like other class I PI3Ks, PI3Kγ stimulates activation of signaling proteins, such as AKT and BTK (Bruton tyrosine kinase), that bear pleckstrin homology, FYVE, and PX domains [1–5]. PI3Kγ enables tumor growth by promoting immune-suppressive myeloid cell trafficking and gene expression [7,10]. This enzyme has thus emerged as a novel myeloid cell target for cancer immune therapy (Figure 2, Key figure), and inhibitors of it have been evaluated in several clinical trials for solid tumor therapy. Because its expression is largely restricted to myeloid cells and endothelial cells, PI3Kγ inhibitors were shown to be safe and nontoxic, with no dose-limiting toxicities in animal models [7,10] and minimal toxicities in patients [17].

PI3Kγ has also recently emerged as a key target for the inhibition of leukemia stemness in acute myeloid leukemia (AML) and other hematogenous cancers [18–24]. Additional roles in the control of myeloid cell and tumor biology demonstrate that this kinase is a key player in tumor biology, infectious diseases, and autoimmunity. PI3Kγ inhibitors thus have growing potential to serve as effective cancer therapeutics. Continued study of the molecular and cellular bases for PI3Kγ roles in cancer will undoubtedly unlock additional clinical applications for PI3Kγ inhibitors.

## PI3Kγ control of myeloid cell adhesion and migration

The gamma isoform of the PI3K family was first identified in 1996 as a phosphatidylinositol kinase activity that was activated by GPCRs and/or by Ras proteins [25–30]. PI3Kγ includes two different heterodimeric variants: PI3Kγ (p84/87) and PI3Kγ (p101). These two variants share the same p110γ catalytic subunit but differ in their associated noncatalytic subunit. The PI3Kγ-p101 complex is activated by GPCRs; Gβγ subunits bind to the p101 adaptor protein of PI3Kγ, enhancing p110γ lipid kinase activity. PI3Kγ p84/p87–p110γ complexes can be activated by RTKs and are activated in a RAS-GTP–dependent manner [7,30]. Once activated, PI3Kγ transfers the γ-phosphate of ATP to phosphatidylinositol(4,5)bisphosphate to produce PIP_3_, which serves as a docking site for effector proteins. PI3Kγ also functions as a scaffold for phosphodiesterase 3B (PDE3B) and thereby inhibits accumulation of cAMP and inhibition of protein kinase A activation, thus regulating cardiomyocyte functions. This scaffolding function of PI3Kγ with PDE3B was elegantly shown by studying PI3Kγ signaling in genetically engineered mice that either completely lacked PI3Kγ expression or expressed a kinase-dead variant [31]. This same scaffolding function promotes phagocytosis in microglia [32].

The migration of neutrophils and macrophages in response to inflammatory stimuli during disease, as well as the neutrophil respiratory burst, require PI3Kγ [7,11,12]. PI3Kγ is required for directional migration and chemotaxis of myeloid cells by coordinating the localization of AKT with F-actin at the plasma membrane [12]. Activation of neutrophils by the bacterial peptide fMLP (*N-formylmethionine-leucyl-phenylalanine*) triggered not only neutrophil chemotaxis but also p47phox-mediated reactive oxygen species production, thereby arming neutrophils with pathogen-seeking and killing capabilities [12]. Recent analyses also showed that PI3Kγ promotes noncanonical, gasdermin D pyroptosis-dependent NETosis, an innate immune mechanism by which neutrophils extrude DNA and enzymes to entrap and kill pathogens, including parasites such as *Leishmania* and during tissue repair [33]. Key studies additionally documented a role for PI3Kγ in the control of macrophage and microglial phagocytosis, an important mechanism by which macrophages clear pathogens and damaged tissues [15,32]. Taken together, these and other studies demonstrate that PI3Kγ controls major pathways by which myeloid cells participate in innate immunity.

PI3Kγ roles in immunity are not limited to infectious diseases, however; this enzyme also plays key roles in promoting tumor growth. Insightful studies showed that endothelial cell PI3Kγ promotes virally induced tumor growth; these researchers demonstrated that a GPCR-encoded by herpesvirus exploits endothelial cell PI3Kγ to promote Kaposi’s sarcoma, an endothelial cell tumor that develops in immune-suppressed patients [9]. These were the first studies to show that PI3Kγ can directly promote tumor growth in some conditions.

**Figure 3.**
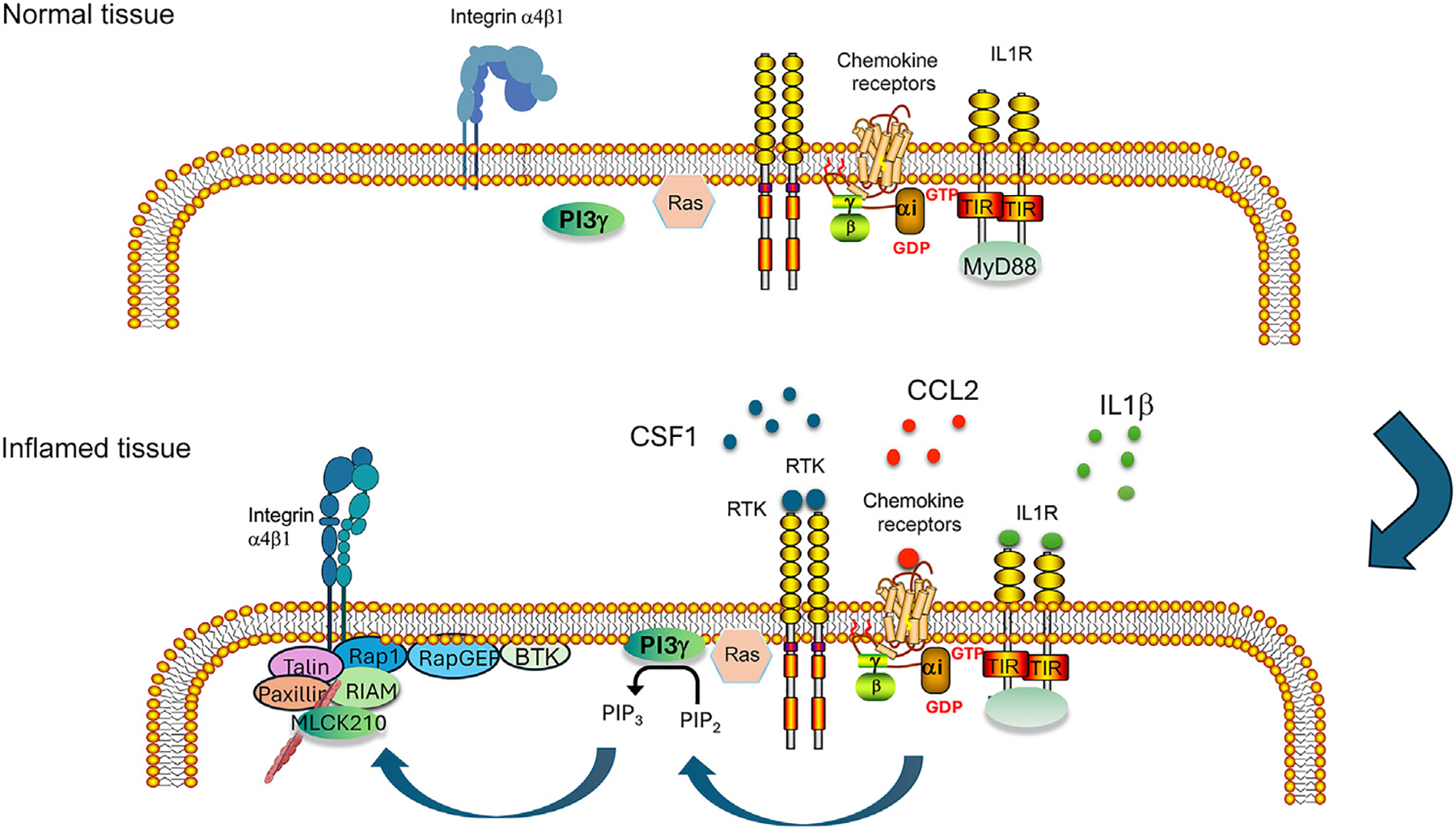
Phosphoinositide 3-kinase-γ (PI3Kγ) promotes myeloid cell trafficking during inflammation. PI3Kγ can be activated by Ras and Gβγ complexes that are associated with G protein–coupled receptors such as CCR2, cytokine receptors such as colony-stimulating factor receptor 1 (CSF1R), and interleukin (IL) receptors such as IL-1R. Once activated, PI3Kγ produces phosphatidylinositol-3,4,5-trisphosphate (PIP_3_) on the inner leaflet of the lipid bilayer. Proteins with pleckstrin homology domains, FYVE domains, or PX domains interact with PIP_3_ to associate within signaling complexes at the plasma membrane. These include the GTPase Rap1, the Rap1-GTP–interacting adapter molecule (RIAM), and RapGEFs. This complex promotes the association of RIAM with Talin, a large integrin binding protein that together with paxillin and a high-molecular-weight form of myosin light chain kinase, cause conformational changes in integrins, particularly integrin α_4_β_1_, leading to integrin activation, cell adhesion, and myeloid cell recruitment to damaged tissues. Abbreviations: BTK, Bruton tyrosine kinase; RTK, receptor tyrosine kinase.

A critical role for PI3Kγ in tumor immune suppression was first described in 2011 [7]. These studies demonstrated that PI3Kγ activated by GPCRs or RTKs within myeloid cells promotes integrin α_4_β_1_-mediated trafficking of immune-suppressive circulating monocytes and granulocytes (MDSCs) into tumors. By inducing integrin conformational changes that stimulate cell adhesion to vascular cell adhesion molecule on endothelial cells, PI3Kγ is required for myeloid cell trafficking into solid tumors, including breast, pancreas, head and neck, melanoma, and other tumors [7,34–36]. Blockade of PI3Kγ with deletion mutants or a PI3Kγ selective inhibitor suppressed angiogenesis, tumor growth, and metastasis in a myeloid cell–dependent manner in genetically engineered mouse models of breast cancer, pancreatic cancer, and other tumors [7,10,34–36]. Further studies showed that H- or K-Ras activation by GPCRs, cytokine receptors, or interleukin (IL) receptors promoted PI3Kγ-mediated Rap1a activation and subsequent Rap-mediated nucleation of Talin, paxillin, and myosin light chain kinase on integrin cytoplasmic tails [7,35,36]. This molecular complex exerts physical stresses on integrin cytoplasmic tails that promote rapid conformational changes in integrins that allow these adhesion proteins to unfold and promote trafficking into tumor tissues (Figure 3). Together, these initial studies of the role of PI3Kγ in cancer showed that deletion of PI3Kγ or antagonists of PI3Kγ suppress tumor growth by reducing recruitment of myeloid cells to tumors.

## PI3Kγ and lipid-associated macrophages

Research from the metabolism, cancer, and immunology fields has identified essential roles for lipid-associated macrophages (LAMs) in homoeostasis and disease, including cancer. LAMs play important roles in the control of lipid homeostasis but also in immune suppression. Lipid uptake in adipose tissue is regulated by LAMs; loss of the lipid uptake receptor Trem2 disrupts lipid homeostasis leading to adipocyte hypertrophy and weight gain [4,37]. Dysregulation of lipid uptake in macrophages also promotes tumor growth; tumor-associated macrophages (TAMs) are characterized by increased fatty acid oxidation, which upregulates PI3Kγ and the subsequent immune-suppressive macrophage phenotype. Inhibition of PI3Kγ with the inhibitor IPI-549 restored macrophage functions and stimulated cytotoxic T cell–mediated tumor regression in a mouse model of LAM-rich gastric cancer [38]. PI3Kγ also regulates signaling by CD36, a long-chain fatty acid transporter, promoting the recruitment of immune-suppressive bone marrow–derived macrophages during liver metastasis [39]. This enzyme also controls lipid uptake into foam cells, macrophages found in blood vessels, lungs, and other tissues by PI3Kγ-mediated macropinocytosis [40].

## PI3Kγ roles in immune suppression: control of transcription and phagocytosis

**Figure 4.**
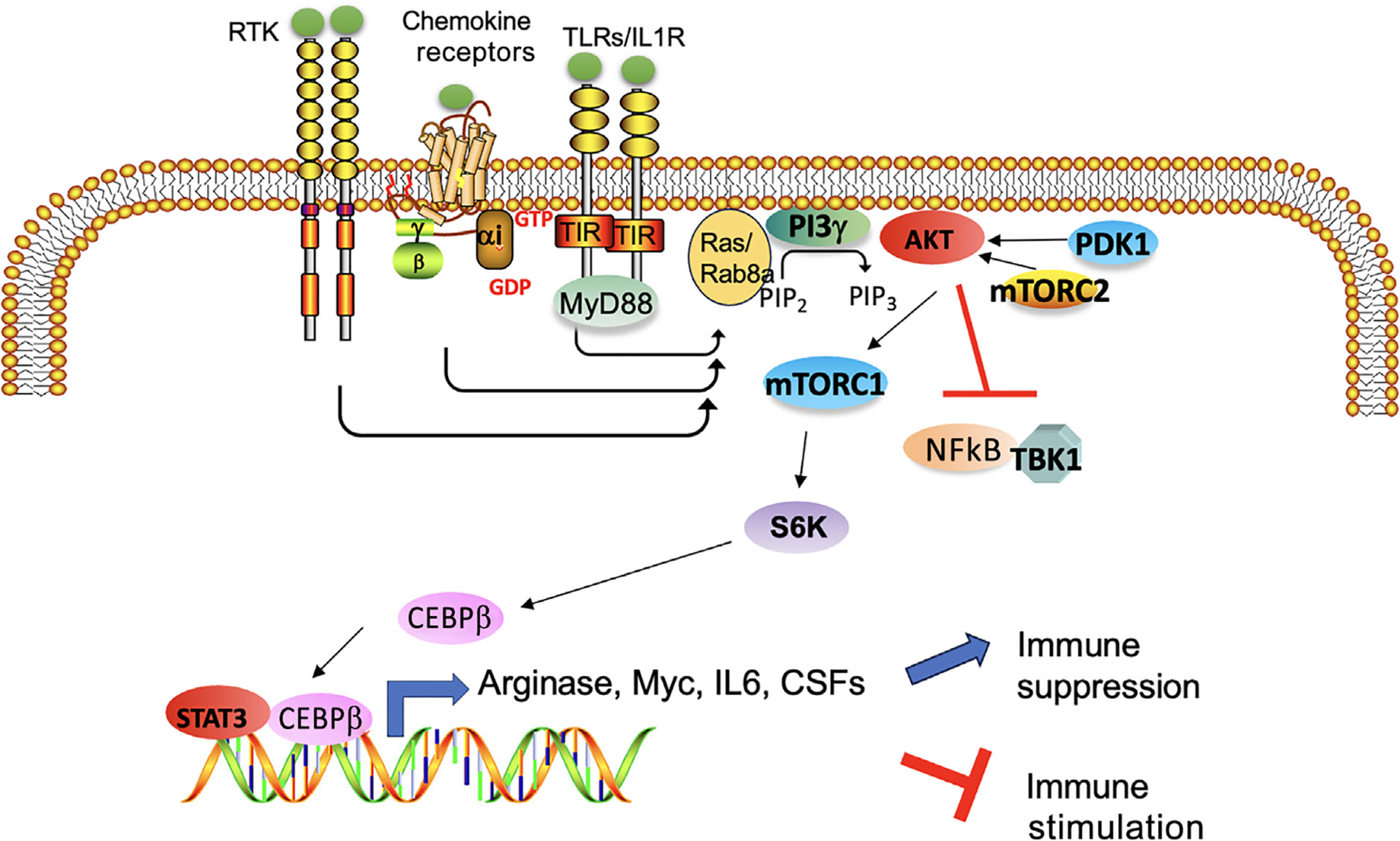
Phosphoinositide 3-kinase-γ (PI3Kγ) modulation of immune-suppressive transcription in myeloid cells. PI3Kγ can be activated by Ras or Rab8a and Gβγ complexes when G protein–coupled receptors, cytokine receptors, interleukin (IL) receptors, and Toll-like receptors are activated by their ligands. Once activated, PI3Kγ produces phosphatidylinositol-3,4,5-trisphosphate (PIP_3_) on the inner leaflet of the lipid bilayer. Proteins such as PDK1 and AKT with pleckstrin homology domains interact with PIP_3_ to associate with the plasma membrane and undergo activation. Once activated, AKT inhibits nuclear factor (NF)-κB/TBK1 and activates mTORC1 (mammalian target of rapamycin complex 1), leading to S6 kinase-dependent activation of C/CAAT enhancer–binding protein beta (CEBPβ), a transcription factor that associates with STAT3 to promote transcription of immune-suppressive factors such as arginase, Myc, IL-6, and colony-stimulating factors (CSFs). PI3Kγ thus promotes immune suppression by blocking proinflammatory cytokine expression and promoting the expression of immune-suppressive cytokines and proteins, such as arginase.

Tumor-associated myeloid cells, including TAMs, MDSCs, and regulatory dendritic cells, exhibit immune-suppressive properties. These cells prevent T cell activation by expressing immune checkpoints, cytokines such as IL-6 family members, IL-10, TGF-β (transforming growth factor-β), and extracellular matrix proteins, such as osteopontin, fibronectin, and even collagen, that exclude T cells from the immediate tumor microenvironment or that directly inhibit T cell viability [41]. Growing evidence has demonstrated that PI3Kγ acts as a **polarization** rheostat that suppresses inflammation and promotes tumor immune suppression (Figure 4).

Shortly after their identification, the PI3Ks were found to negatively regulate cytokine production in macrophages and dendritic cells [42]. Several years later, this activity was attributed to downregulation of Toll-like receptor (TLR)-induced proinflammatory myeloid signaling and cellular responses by the class IB isoform PI3Kγ [10,43]. TLRs are specialized cell surface and endosome receptors that respond to pathogen-associated molecular patterns; foreign sequences from bacteria, viruses, and parasites; and damage-associated molecular patterns from damaged tissues by promoting nuclear factor (NF)-κB–dependent inflammatory cytokine expression. A series of publications have demonstrated that PI3Kγ is activated by an Rab8a/TLR complex, which then activates Akt/mammalian target of rapamycin (mTOR) to suppress proinflammatory cytokine expression [43–45]. Moreover, PI3Kγ-mediated suppression of inflammatory cytokine expression promotes tumor growth [10,46–52]; these effects could be reversed by PI3Kγ deletion or inhibition using PI3Kγ or mTOR inhibitors [10,47,49,50]. Selective inhibition of PI3Kγ promotes effective antitumor immune responses [10,47–52]. These studies showed that PI3Kγ signaling through Akt and mTOR inhibits NF-κB activation but stimulates C/CAAT enhancer–binding protein beta (C/EBPβ) activation, thereby inducing a transcription program that promotes immune suppression during inflammation and tumor growth [10]. Inactivation of macrophage PI3Kγ prolonged NF-κB activation and inhibited C/EBPβ activation, thereby stimulating an immunostimulatory transcription program that recruits CD8^+^ T cells and stimulates CD8 T cell–dependent cytotoxicity [10]. These and other studies showed that PI3Kγ inhibition synergizes with **checkpoint inhibitors** in animal models of cancer to promote tumor regression and increased survival [10,47]. Studies in mouse models of pancreatic cancer showed that myeloid cell PI3Kγ also drives macrophages to promote tumor cell invasion, metastasis, and desmoplasia in pancreatic ductal adenocarcinoma (PDAC) [49,50] in part by controlling macrophage expression of PDGF (platelet-derived growth factor) and subsequent tumor cell migration and fibroblast production of collagen [49]. Blockade of PI3Kγ in PDAC-bearing mice reprogrammed TAMs to inhibit tumor cell invasion, metastasis, and desmoplasia [49,50]. These data indicated the central role that macrophage PI3Kγ plays in controlling tumor fate. PI3Kγ can also promote tumor stemness by controlling IL-6 family member expression by macrophages; pharmacologic inhibition or genetic inactivation of PI3Kγ disrupted IL-6 family member signaling in tumor cells and reduced tumor growth in mouse models of glioblastoma [52]. PI3Kγ inhibition also suppresses carcinogen-induced tumor development [53]. PI3Kγ inhibition can synergize with other forms of immune therapy. For example, PI3Kγ inhibition synergized with an α-enolase (ENO1) DNA vaccination to counteract PDAC tumor growth, reducing associated stroma and T cell exhaustion and increasing follicular helper T cell activation and ‘antigen spreading,’ because many other tumor-associated antigens were recognized by IgG2c ‘cytotoxic’ antibodies [54]. Importantly, PI3Kγ inhibition enhances dendritic cell cross-presentation [55]. Taken together, these studies highlighted the translational potential for PI3Kγ inhibition as a therapy for numerous solid tumors.

Several PI3Kγ inhibitors have been developed and investigated in preclinical settings; several have been investigated in the clinic (Table 1). The PI3Kγ inhibitor IPI-549, eganelisib, was developed as a novel immune therapeutic targeting myeloid cells and has been evaluated in several phases I and II clinical trials as a cancer therapeutic [17,56]. Eganelisib exhibited minimal dose-limiting toxicities and showed some promise as a therapeutic when combined with checkpoint inhibitors and chemotherapy [17,56]. Results in phase II trials confirmed that PI3Kγ inhibition repolarizes myeloid cells and enhances T cell responses [56]. Pre- and on-treatment paired tumor biopsies from a phase II trial of eganelisib + nab-paclitaxel + atezolizumab (anti–PD-L1) in patients with triple-negative breast cancer displayed evidence of myeloid cell reprogramming, T cell activation, and extracellular matrix reorganization. Gene signatures of immune activation, including expression of T cell recruiting chemokines such as CXCL10 and decreased SPP1 (osteopontin) were observed in patients with prolonged progression-free survival. Peripheral blood analyses revealed gene expression signatures of systemic immune activation, with increased gene expression signatures of MHCI and MHCII antigen presentation and T cell activation [56]. These studies indicate that PI3Kγ inhibitors hold promise as therapeutics for solid tumors.

## Roles of PI3Kγ in leukemias

Very recently, PI3Kγ has emerged as an actionable therapeutic target in leukemias and lymphomas. Using shRNA knockdowns and selective inhibitors, PI3Kγ and PI3Kδ were both found to control AML and mantle cell lymphoma, with PI3Kδ regulating proliferation and PI3Kγ regulating migration of these aggressive tumor types [18,19]. The dual δ/γ inhibitor IPI-145 (duvelisib) controlled proliferation and migration of AML and mantle cell lymphoma cells *in vitro* and *in vivo*, suggesting its usefulness in the treatment of these aggressive diseases [18,19]. PI3Kγ also promotes anaplastic lymphoma kinase (ALK)-driven anaplastic large cell lymphoma (ALCL). Identification of a survival pathway activated by the chemokine receptor 7 (CCR7) and PI3Kγ signaling [20] suggested that a constitutively active PI3Kγ isoform cooperates with oncogenic ALK to accelerate lymphomagenesis in mice. Blockade of PI3Kγ or CCR7 together with ALK tyrosine kinase inhibitors reduced ALK inhibitor resistance and the survival of lymphoma cells in ALCL [20].

The PI3Kδ/γ inhibitor duvelisib was approved for clinical use in the treatment of relapsed or refractory chronic lymphocytic leukemia (CLL) in 2018. However, serious adverse events, including colitis, pneumonitis, and anaphylaxis, sometimes occurred, prompting the FDA to issue a black box warning. An explanation for these effects came from studies which showed that selective inhibition of PI3Kδ or PI3Kγ could suppress T cell proliferation; dual inhibition of PI3Kγ and PI3Kδ with duvelisib was found to most potently inhibit T cell proliferation [21]. It must be noted, however, that the effects of PI3Kγ inhibition on T cell proliferation are unclear; many reports indicate that PI3Kγ does not affect T cell proliferation, and serious side effects of PI3Kγ inhibitors have not been observed [10,17,56]. Taken together, these observations showed that PI3Kγ might be a useful clinical target for the treatment leukemias and lymphomas.

**Figure 5.**
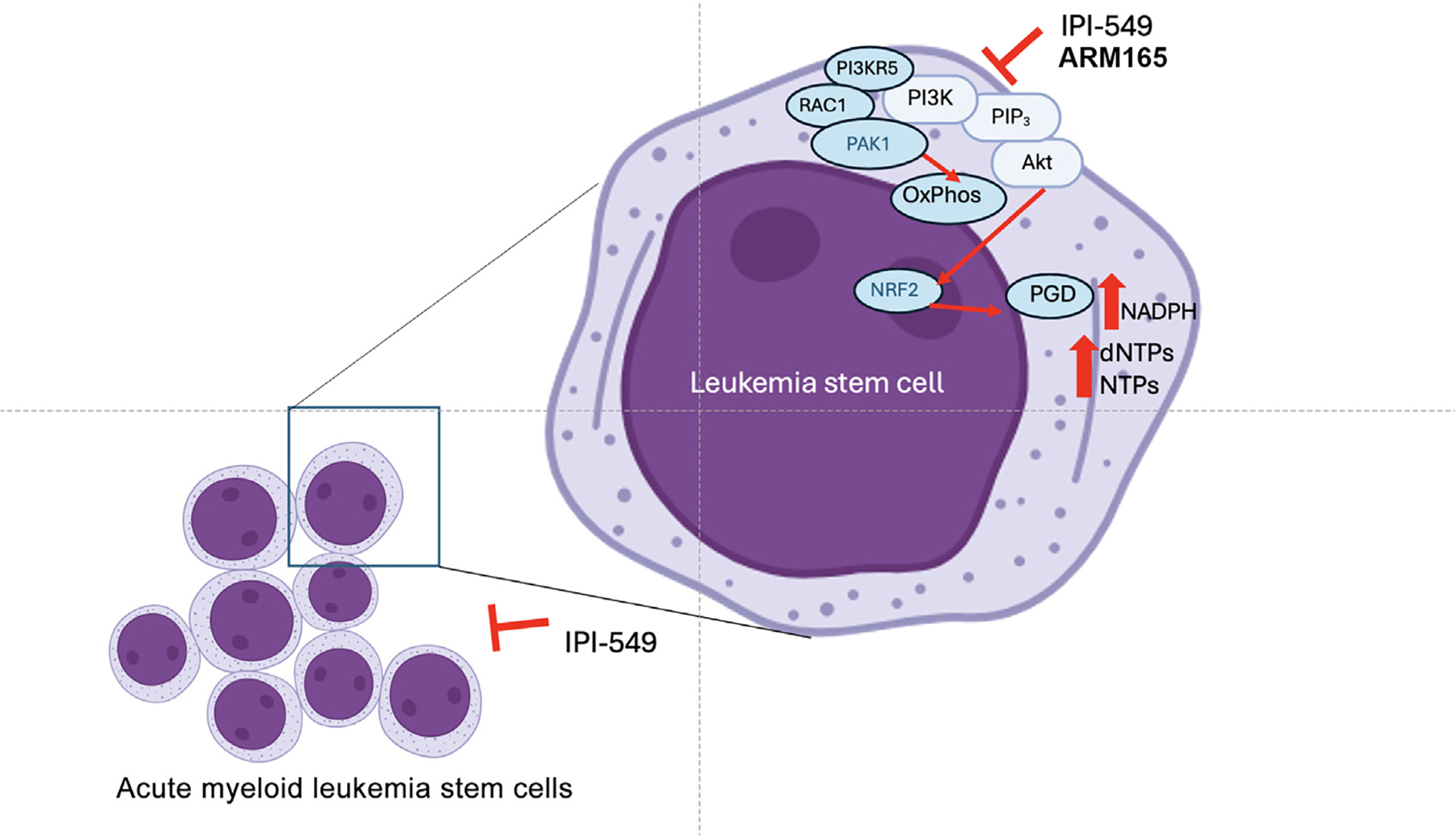
Control of acute myeloid leukemia (AML) stemness by phosphoinositide 3-kinase-γ (PI3Kγ). PI3Kγ promotes leukemic stem cell self-renewal and proliferation by regulating leukemic cell metabolism. PI3Kγ-mediated activation of AKT activates nuclear factor erythroid 2-related factor 2 (NRF2) and promotes its translocation to the nucleus, where it promotes transcription of 6-phosphogluconate dehydrogenase (PGD) and the pentose phosphate pathway, thereby stimulating AML stemness. Additionally, PI3Kγ activates p21-activated kinase (PAK1), which promotes oxidative phosphorylation and leukemia progression. IPI-549, the clinical stage PI3Kγ inhibitor, and ARM165, a PI3Kγ PROTAC, suppress these events and block AML self-renewal and progression in animal and human models of disease.

Further evidence for the essential roles of PI3Kγ in leukemia appeared in a series of publications in 2024 (Figure 5). PI3Kγ was identified as a key regulator of AML stemness [22]. Studies of AML cells revealed that PI3Kγ is highly enriched in leukemia stem cells (LSCs) and was required for self-renewal of AML cells, but not for self-renewal of normal hematopoietic stem cells. PI3Kγ-AKT signaling promotes nuclear factor erythroid 2-related factor 2 (NRF2) nuclear accumulation and subsequent expression of 6-phosphogluconate dehydrogenase, a key member of the pentose phosphate pathway, which maintains LSC stemness [22]. Furthermore, inhibition of the PI3Kγ pathway eliminated LSCs without damaging normal hematopoiesis, providing a promising therapeutic strategy for AML [22]. A concurrent study used a genome-wide CRISPR screen of acute leukemias to show that PI3Kγ and its regulatory subunit PI3K regulatory subunit 5 (PIK3R5, p101) are both required for acute leukemia progression [23]. Treatment with the selective PI3Kγ inhibitor eganelisib was effective in inhibiting the growth of leukemias with elevated PIK3R5 (p101) in a noncanonical p21-activated kinase (PAK1)-dependent manner; a combination of eganelisib and cytarabine prolonged AML survival compared with single-agent therapy [23]. A third study published in 2024 demonstrated association of high expression of PIK3CG and PIK3R5 and resistance to venetoclax, a standard-of-care BCL2 inhibitor used to treat human AML. These studies showed that ARM165, a proteolysis-targeting chimera (PROTAC) of AZ2, a selective PI3Kγ inhibitor, specifically degraded PI3Kγ and suppressed AML progression [24]. When combined with venetoclax, this PROTAC inhibited leukemia progression in cell lines, primary AML cells, and syngeneic mouse models of leukemia [24]. Addition of the protein degrader function to AZ2 improved the efficacy of this PI3Kγ selective inhibitor, suggesting that PI3Kγ degradation may be a more effective strategy for the treatment of AML than inhibition of the catalytic function of this enzyme. Together, these recent studies show that PI3Kγ is a valuable target in oncology, both for solid tumors and for leukemias.

## PI3Kγ in other disease settings: insights for cancer therapy

A subset of cancers is virally induced, including cervical cancer, some head and neck cancers, liver cancer, and some lymphomas. An understanding of the role of PI3Kγ in infectious diseases could benefit cancer prevention. Recent studies during the COVID-19 epidemic investigated the impact of PI3Kγ on severe acute respiratory syndrome coronavirus 2 (SARS-CoV-2) and other coronavirus infections and subsequent inflammation. Although these studies found that PI3Kγ did not control virus uptake or infectivity, PI3Kγ promoted myeloid cell–mediated inflammation and tissue damage associated with viral infection. Tissue-damaging inflammation and vascular leak associated with SARS-CoV-2 were ameliorated with inhibitors of PI3Kγ [16]. PI3Kγ inhibition also reduced vascular leak, thrombocytopenia, liver damage, cytokine storm, and viral replication associated with dengue infection [57]. Infectivity by polyomaviruses was also suppressed by PI3Kγ inhibition; however, because PI3Kγ inhibition did not suppress virus binding or internalization, it is not yet clear how it regulates infectivity [58].

Myeloid cells play key roles in wound healing; their excessive wound-healing responses can result in fibrosis that disrupts the proper functioning of delicate tissues. For example, prolonged use of ventilators can lead to long-term lung damage and fibrosis. PI3Kγ inhibition can ameliorate fibrosis and lung damage in response to ventilator-induced injury or bleomycin administration by suppressing pulmonary inflammation [59–61]. PI3Kγ inhibition ameliorates disease progression and symptoms in experimental autoimmune encephalomyelitis, a mouse model of the autoimmune disorder multiple sclerosis [62,63], and can reduce autoantibody production in a mouse model of lupus [64]. Additionally, PI3Kγ inhibitors show efficacy in suppressing asthma because they reduce eosinophil recruitment to airways [65]. PI3Kγ also plays direct roles in cancer cells; two recent studies documented key roles for PI3Kγ in the control of macropinocytosis in tumor cells [66,67]. These studies show that by promoting macropinocytosis, PI3Kγ facilitates tumor escape from autophagy inhibition.

## PI3Kγ targeted therapeutic approaches

Several selective PI3Kγ inhibitors have been developed since the discovery of the enzyme almost 25 years ago. Efforts to synthesize highly selective, orally active inhibitors continue with novel chemical platforms and application of machine learning methods [68,69]. Interesting new approaches include development of nanoparticles containing PI3Kγ inhibitors, such as those that are coloaded with STING agonists and PI3Kγ inhibitors in a ferric ion–punicalagin network (Fe-PU), an inducer of ferroptosis [70], or albumin nanoparticles containing a PI3Kγ inhibitor and paclitaxel for breast cancer therapy [71]. Inhibitors that have been developed and extensively tested in animal models or in the clinic are shown in Table 1. The earliest PI3Kγ inhibitors developed by Ares-Serono, AS252424 and AS605240, were extensively tested in mouse models of asthma, arthritis, and lupus and were not completely selective for PI3Kγ [72–74]. The first PI3Kγ inhibitor to be tested in the clinic was TG100–115, developed by TargeGen for the inhibition of ischemia-reperfusion damage associated with myocardial infarction and vascular leak [75,76]. The PI3Kδ/γ inhibitor duvelisib, or IPI-145, was developed by Infinity Pharmaceuticals, Verastem Oncology, and Secura Bio for the treatment of CLL and other leukemias and lymphomas. Approval for the treatment of CLL was granted in 2018 [77,78]. However, significant treatment-related fatalities were associated with the use of duvelisib, promoting caution regarding the use of this agent [79]. A current clinical trial is underway testing the combination of duvelisib and anti–PD-1 for solid tumor therapy (Table 1). IPI-549 (eganelisib), a highly selective PI3Kγ inhibitor, was developed by Infinity Pharmaceuticals and evaluated in several phases I and II clinical trials with evidence of improved antitumor immune responses [17,46,56]. AstraZeneca developed AZD8154, a PI3Kδ/γ inhibitor that was tested in a phase I clinical trial, as well as AZD3458, a PI3Kγ inhibitor that was tested in animal models of cancer [48,65,80]. Nanjing Zenshine Pharmaceuticals is testing ZX-4081, a highly selective PI3Kγ inhibitor, in solid tumor clinical trials [81]. On the basis of recent observations that PI3Kγ regulates AML stemness, future clinical trials testing PI3Kγ inhibitors for AML are in the planning stages.

## Concluding remarks and future perspectives

PI3Ks play pivotal roles in cancer biology, from serving as oncogenes to promoting tumor immune suppression. The unique class IB PI3Kγ controls immune responses in normal myeloid cells and stemness in myeloid tumor cells. This lipid kinase thus plays pivotal roles in controlling myeloid cell recruitment to tumors as well as immune-suppressive transcription in the tumor microenvironment. Highly selective PI3Kγ inhibitors have demonstrated safety and therapeutic efficacy in animal models of solid tumors and leukemias. Clinical trials testing PI3Kγ inhibitors have shown some promise for the treatment of solid tumors, particularly in combination with checkpoint inhibitors and chemotherapy. New studies have shown that this class of drugs can control AML as well. Although key questions remain about which PI3Kγ roles are most important in oncology and which therapeutic approaches targeting PI3Kγ will be most effective (see Outstanding questions), it is likely that PI3Kγ inhibitors will continue to show synergy with new therapeutic approaches, including chimeric antigen receptor T cells, chemotherapy, radiation, and cancer vaccines, for both solid tumors and leukemias.

## Figures and Tables

**Table 1.** PI3Kγ inhibitors: preclinical and clinical studies

PI3Kγ inhibitor	Company	Selectivity (kinase assay IC_50_)	Stage	Animal models evaluated	Clinical indication	Clinical trial name/number	Refs
AS252424	Ares-Serono	PI3Kγ 30 nM PI3Kδ 20uM PI3Kα 940 nM PI3Kβ 20μM	Preclinical	Asthma	N/A	N/A	[72]
AS605240	Ares-Serono	PI3Kγ 8 nM PI3Kδ 300 nM PI3Kα 60 nM PI3Kβ 270 nM	Preclinical	Arthritis, lupus	N/A	N/A	[73,74]
TG100–115	TargeGen	PI3Kγ 83nM PI3Kδ 243 nM PI3Kα 1.2 μM PI3Kβ 1.3μM	Clinical	Vascular leak ischemia/reperfusion Solid tumors	Phase I ischemia-reperfusion/myocardial infarction	NCT00103350 ^i^	[7,8,10,34–36,49,50,52,75,76]
Duvelisib IPI-145	Infinity Pharmaceuticals/Verastem Oncology/Secura Bio	PI3Kγ 27 nM PI3Kδ 2.5 nM PI3Kα 1602 nM PI3Kβ 85 nM	Clinical, approved 2018	Arthritis, asthma, lupus, lymphoma, solid tumors	Phase III (approved) CLL/SLL	NCT02004522 ^ii^	[77,78]
Duvelisib IPI-145	Verastem Oncology; CSPC ZhongQi Pharmaceutical Technology Co.	PI3Kγ 27 nM PI3Kδ 2.5 nM PI3Kα 1602 μM PI3Kβ 85 nM	Clinical	Arthritis, asthma, lupus, lymphoma, solid tumors	Phase I/II advanced solid tumors	NCT05508659^iii^ with recombinant anti-PD-1 (SG001)	[77,78]
IPI-549 (Eganelisib)	Infinity Pharmaceuticals	PI3Kγ 16 nM PI3Kδ>8400 nM PI3Kα 3200 nM PI3Kβ 3500 nM	Clinical	Solid tumors, acute myeloid leukemia, SARS-CoV-2	Phase I/Ib advanced solid tumors Phase II urothelial cancer Phase II triple-negative breast cancer Phase II metastatic triple-negative breast cancer/ovarian cancer Phase II head and neck cancer	MARIO-1 NCT02637531^iv^ with nivolumab MARIO-275 NCT03980041^v^ with nivolumab MARIO-3 NCT03961698^vi^ with nab-paclitaxel + tecentriq NCT03719326^vii^ with etrumadenant (AB928) + pegylated liposomal doxorubicin or etrumadenant + nab-paclitaxel NCT03795610^viii^ window of opportunity study	[10,16,17,46,47,56]
AZD8154	AstraZeneca	PI3Kγ 0.76 nM PI3Kδ 4.3 nM PI3Kα >18.4 μM PI3Kβ > 30 μM	Clinical	Asthma	Phase I healthy Volunteers	NCT03436316 ^ix^	[65,80]
AZD3458	AstraZeneca	PI3Kγ 7.9 nM PI3Kδ 1 μM PI3Kα 31 μM PI3Kβ 31 μM	Preclinical	Clinical, phase I	N/A	N/A	[48]
ZX-4081	Nanjing Zenshine Pharmaceuticals	PI3Kγ 1.5 nM PI3Kδ >1.5 μM PI3Kα >1.5 μM PI3Kβ >1.5 μM	Clinical, phase I	Solid tumors	Phase I advanced solid tumors	NCT05118841 ^x^	[81]

## Highlights

Phosphoinositide 3-kinase-γ (PI3Kγ) controls membrane dynamics to promote integrin activation, chemotaxis, macropinocytosis, phagocytosis, and tissue remodeling.

Trafficking of monocytes and granulocytes to tumors, inflamed tissues, and sites of infection requires PI3Kγ.

PI3Kγ restrains inflammation via AKT/mammalian target of rapamycin–mediated repression of immune response transcription in myeloid cells.

Neutrophil net formation and vascular leak depend on PI3Kγ.

PI3Kγ inhibition suppresses solid tumor growth, desmoplasia, and metastasis.

PI3Kγ promotes myeloid leukemia self-renewal.

## Outstanding questions

Which roles of PI3Kγ are most important in solid tumors and in hematogenous tumors?

What are the optimal therapeutic and timing strategies to combine with PI3Kγ-targeted therapeutics in solid and hematogenous tumors?

Does PI3Kγ inhibition have dose-limiting toxic side effects alone or in combination with other therapeutics, and what is the cellular and molecular cause of these toxicities?

What is the best approach to inhibit PI3Kγ roles in cancer? Will current small-molecule inhibitors, encapsulated inhibitors, PROTACS, covalent inhibitors, gene expression modifiers, or inhibition of downstream targets be most effective at controlling myeloid cell–mediated immune suppression and roles in promoting AML stemness?

## Glossary

Checkpoint inhibitors: antibodies directed against checkpoints, cell surface proteins on T cells, tumor cells, or antigen-presenting cells that suppress T cell activation and prevent immune system overactivation.
Immune suppression: inhibition of innate and adaptive immune system recognition and response to foreign antigens and cancer.
NETosis: the process by which neutrophils extrude DNA, histones, and enzymes in tissues.
Neutrophil extracellular traps: networks of fibers composed of DNA, histones, and enzymes extruded from neutrophils to bind and kill pathogens.
PI3Ks: lipid kinases that phosphorylate phosphatidylinositol in the 3′ position to control activation of proteins expressing pleckstrin, FYVE, or PX domains.
PI3K class I isoforms: the class I PI3Ks α, β, γ, and δ were the first PI3Ks to be described. These kinases act at the plasma membrane and are activated by G protein–coupled receptors (GPCRs), receptor tyrosine kinases (RTKs), cytokine receptors, and Toll-like receptors (TLRs).
PI3Kγ: a class IB PI3K, PI3Kγ is a lipid kinase primarily expressed in myeloid cells, vascular endothelial cells, and some tumor cells, where it regulates immune responses, vascular permeability, and stemness.
Polarization: shifts in cell shape and gene expression patterns in response to extracellular stimuli.
Trafficking: directed migration of immune cells into tissues, including tumors, that depends on PI3K signal transduction processes that activate integrins to facilitate cell adhesion to and movement through endothelial cells.
